# Clinical diagnosis versus autopsy findings in polytrauma fatalities

**DOI:** 10.1186/1757-7241-18-55

**Published:** 2010-10-26

**Authors:** Claas T Buschmann, Patrick Gahr, Michael Tsokos, Wolfgang Ertel, Johannes K Fakler

**Affiliations:** 1 University Medical Centre Charité - University of Berlin, Institute of Legal Medicine and Forensic Sciences, Turmstr. 21, Building L, 10559 Berlin, Germany; 2 University Medical Centre Charité - University of Berlin, Department of Orthopedic, Trauma and Reconstructive Surgery, Campus Benjamin Franklin, Hindenburgdamm 30, 12200 Berlin, Germany; 3 MediClin Waldkrankenhaus Bad Düben, Hospital for Special Orthopedic Surgery, Gustav-Adolf-Straße 15a, 04849 Bad Düben, Germany

## Abstract

**Objectives:**

The aim of the study was to determine if differences in clinical diagnosis versus autopsy findings concerning the cause of death in polytrauma fatalities would be detected in 19 cases of fatal polytrauma from a Level 1 trauma centre.

**Methods:**

Clinical diagnoses determining the cause of death in 19 cases of fatal polytrauma (2007 - 2008) from a Level 1 trauma centre were correlated with autopsy findings.

**Results:**

In 13 cases (68%), the clinical cause of death and the cause of death as determined by autopsy were congruent. Marginal differences occurred in three (16%) patients while obvious differences in interpreting the cause of death were found in another three (16%) cases. Five fatalities (three with obvious differences and two with marginal differences) were remarked as early death (1-4 h after trauma) and one fatality with marginal differences as late death (>1 week after trauma). Obvious and marginal discrepancies mostly occurred in the early phase of treatment, especially when severely injured patients were admitted to the emergency room undergoing continued cardiopulmonary resuscitation, i. e. limiting diagnostic procedures, and thus the clinical cause of death was essentially determined by basic emergency diagnostics.

**Conclusions:**

Autopsy as golden standard to define the cause of death in fatal polytrauma varies from the clinical point of view, depending on the patient's pre-existing condition, mechanism of polytrauma, necessity of traumatic cardiopulmonary resuscitation, survival time, and thus the possibility to perform emergency diagnostics. An autopsy should be performed at least in cases of early fatal polytrauma to help establishing the definite cause of death. Moreover, autopsy data should be included in trauma registries as a quality assessment tool.

## Introduction

Polytrauma, defined as a trauma pattern with an Injury Severity Score (ISS) >16 points and consisting of various injuries of which at least one is life-threatening, is associated with a mortality rate up to 23% [1,2]. Severe injury is the leading cause of death among children, adolescents, and young adults (ages 1-44), and represents the third most common cause of death for all ages in the western countries, after cardiovascular diseases and cancer [3]. Frequent causes of death in trauma fatalities are at first injuries to the central nervous system (40-50%), followed by hemorrhage (20-40%) and multiple organ failure (MOF), accounting for a further 2-10%. About 53-69% of deaths occur prior to admission to hospital, and 7.3% of polytrauma patients are reported to have received cardiopulmonary resuscitation (CPR) during pre-hospital or emergency room (ER) treatment [2,4].

In the last decades, numerous clinical implementations, scoring systems and guidelines for improving quality of treatment in polytrauma patients such as the Advanced Trauma Life Support^®^-(ATLS)-Program have been set up [2]. In cases of trauma death, review of autopsy data is also used as part of the trauma quality assurance (QA) process, and autopsy rates are queried by the American College of Surgeons Committee on Trauma in their reviews [5]. Generally, autopsy assessment to in- and out-of-hospital fatalities is a valuable contributor to medical education [6], and current literature supports the concept that autopsies are useful in uncovering a potentially missed diagnosis in trauma patients [7-11]. Especially in death after trauma, autopsy data can provide sufficient data to assist in determining the presence of missed injuries contributory to death [12], and also confirming the clinical cause of death. However, recent studies focusing on causes of death after injury are - with few exceptions [11-13] - solely based on clinical records [2-4,14]. Moreover, in recent publications the value of autopsies after severe trauma is discussed to offer only little additional information [5,6], i.e., no major or minor discrepancies, that may have altered the patient's therapy or survival time in terms of a preventable death or missed injuries.

The aim of the present study was to determine if differences in clinical diagnosis versus autopsy findings concerning the cause of death in polytrauma fatalities would be detected in 19 cases of fatal polytrauma from a Level 1 trauma centre. The value of forensic autopsy in polytrauma fatalities, especially in cases of early death and continued CPR on arrival at the ER (when intended diagnostic procedures possibly were not completed at the occurrence of death) is discussed.

## Materials and methods

Our patient collective was excerpted from 29 cases of fatal polytrauma (2007-01-01 - 2008-12-31) from the Department of Orthopedic, Trauma and Reconstructive Surgery at Campus Benjamin Franklin, University Medical Centre Charité - University of Berlin/Germany, a Level I university trauma Center. The total number of polytrauma patients admitted to this hospital during the study period was 174 (mortality rate 16.6%). Polytrauma fatalities that did not have an autopsy performed were excluded.

By order from the public prosecutor's office, 19 of these 29 patients were autopsied at the Institute of Legal Medicine and Forensic Sciences, University Medical Centre Charité - University of Berlin/Germany (autopsy rate 65.5%). Median age was 48 years (24-90 years), 15 patients were male and 4 female. Clinical records, death certificates with date and time of death and final autopsy protocols were evaluated, and then time of death was assigned to 1 of 3 mortality types according to Trunkey's trimodal temporal distribution model [15]. Beyond that, clinical diagnoses determining the cause of death in these 19 fatalities were compared to autopsy findings. For determination of discrepant diagnoses, clinical records were reviewed separately, independently, and retrospectively without knowledge of autopsy findings and vice versa by the authors from two different departments, performing a single case analysis with an interdisciplinary approach. Clinical diagnoses were annotated based on hospital charts and death certificates, and autopsy diagnoses were annotated based on final autopsy reports. Discrepancies were classified in obvious and marginal differences. Obvious discrepancies were defined as autopsy findings that were clinically unsuspected, incorrect or interpreted differently when compared to the clinical records and death certificate, the latter presenting different pathophysiogical pathways contributory to death. Marginal discrepancies were defined as unsuspected or incorrect findings at autopsy when compared to clinical records that did not directly contribute to the patient's death but likely would have had an impact on the patient's treatment or hospital course. Referring to literature, it was questioned whether the patient's death would have been preventable in cases of additional and/or incorrect clinical diagnoses or not [16].

## Results

### Injury patterns

High velocity traffic accidents were the leading cause of polytrauma (n = 11/52%), followed by fall from height (n = 5/26%). Injury patterns were caused by traffic accidents as pedestrian (n = 4) or car-/(motor)cycle driver (n = 7), fall from height (n = 5), homicidal stab attack (n = 1), work place accident (n = 1) and train overrun (n = 1). The median ISS at admission to the ER was scored 45 points (± 15.5 SD), ranging from 33 to 75 points. The stated ISS scorings in this study were solely based on clinical findings.

Traffic accidents caused multiple trauma patterns to the whole body, including severe traumatic brain injury (sTBI), multiple fractures and contusions of extremities and trunk as well as hollow organ and great vessel lacerations; incident-related specific injury patterns were not identified in this group. Falls from height mainly presented sTBI as cause of death; although blunt cardiac injuries have been described to occur frequently at least in falls exceeding >6 m [17], in our small collective such injuries were not diagnosed. Other mechanisms of trauma resulted in corresponding injuries: isolated sharp thorax trauma for homicidal stab attack (n = 1), suicidal train overrun for polytrauma including lower leg amputation (n = 1), and work place accident (patient bruised by an 1800-kg-transformator in a hoistway, n = 1) for blunt polytrauma.

### Cause of death

**Figure 1:**
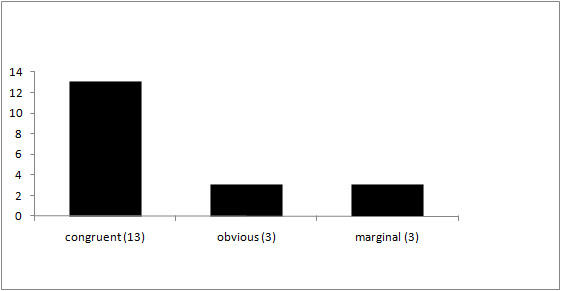
**Comparison of the causes of death before and after autopsy (n = 19)**.

The prevalence of discrepancies in our collective reached 32% (Figure 1), and obvious differences occurred in 3 patients (16%) while marginal differences in interpreting the cause of death were found in another 3 cases (16%). In 13 cases (68%), the clinical cause of death and the cause of death determined by forensic autopsy were congruent, but 2 of these cases (15%) had been explanted for organ donation purposes before autopsy, thus autopsy approved the clinical cause of death, but could not reveal any discrepancies with regard to the explanted organs. None of the fatalities were classified as preventable deaths. A detailed list of the included patients is given in Table 1.

**Table 1:** List of included patients.

no	sex	age	mechanism of polytrauma	ISS	survival time (hh:mm/days if appropriate)	cause of death (clinically)	cause of death (autoptically)	congruent?	CPR	marginal difference	obvious difference
1	f	41	traffic accident (car)	51	2:43	thorax trauma	thorax trauma	**yes**	-	-	-

2	f	24	homicidal stab attack	75	1:00	hemorrhagic shock due to multiple thoracal stab wounds	fatal bleeding due to multiple thoracal stab wounds, bilateral hematopneumothorax	**no - bilateral hematopneumothorax?**	**+**	**+**	-

3	m	46	traffic accident (car)	34	2:15	hemorrhagic shock due to liver rupture, thorax trauma, contusio cordis	thorax trauma, exsanguination from liver rupture	**yes**	-	-	-

4	m	48	traffic accident (car)	50	184 days	MOF/lung artery embolism	MOF	**no - lung artery embolism?**	-	**+**	-

5	f	75	traffic accident (cyclist)	38	15 days	sTBI	sTBI	**yes**	-	-	-

6	m	49	fall from height	54	9:35	sTBI	sTBI	**yes**	-	-	-

7	m	27	traffic accident (car)	59	2:50	sTBI, lung failure, hemorrhagic shock, thorax trauma	sTBI, bilateral lung contusion, pelvic trauma	**no - pelvic trauma ?**	-	-	**+**

8	m	72	traffic accident (pedestrian)	43	2:46	thorax trauma, heart contusion, hemorrhagic shock	thorax trauma, pericardial tamponade	**no - pericardial tamponade?**	**+**	-	**+**

9	m	49	traffic accident (cyclist)	43	9 days	sTBI	sTBI - explanted	**yes - explanted**	-	-	-

10	m	45	train overrun	75	2:22	sTBI/hemorrhagic shock from Sinus sagittalis superior bleeding	sTBI	**no - hemorrhagic shock?**	-	**+**	-

11	m	36	fall from height	38	20:07	sTBI	sTBI	**yes**	-	-	-

12	m	49	work place accident	75	2:10	blunt thoraco-abdominopelvic trauma	fatal bleeding due to pelvic trauma	**no - pelvic trauma?**	**+**	-	**+**

13	m	41	traffic accident (cyclist)	41	1:40	sTBI, blunt abdominal and pelvic trauma	sTBI, blunt abdominal and pelvic trauma	**yes**	-	-	-

14	m	84	fall from height	33	5 days	sTBI	sTBI	**yes**	-	-	-

15	m	57	fall from height	45	7:15	blunt abdominal and pelvic trauma	blunt abdominal and pelvic trauma	**yes**	-	-	-

16	m	33	traffic accident (cyclist)	75	1:10	aortic rupture	aortic rupture	**yes**	**+**	-	-

17	m	45	traffic accident (cyclist)	75	6 days	sTBI	sTBI - explantated	**yes - explanted**	-	-	-

18	m	76	traffic accident (pedestrian)	43	7:46	sTBI	sTBI	**yes**	-	-	-

19	m	95	fall from height	41	21 days	lung artery embolism	lung artery embolism	**yes**	-	-	-

### Survival time and discrepant diagnoses

**Figure 2:**
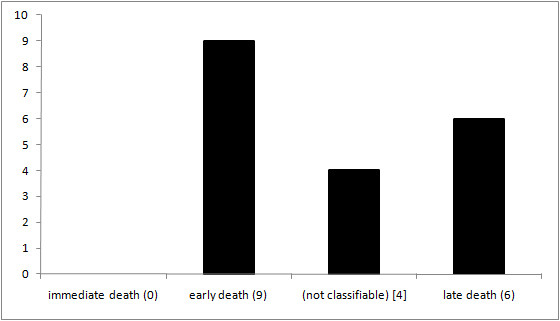
**Survival time according to Trunkey's trimodal temporal distribution model (n = 19)**.

None of the studied fatalities occurred as immediate death (on scene/1-59 min after trauma), conceivably due to the fact that resuscitation procedures at the site of the incident and transport to the ER took more than one hour in our study population. 9 fatalities (47%) occurred as early death (Emergency Room [ER] or Operation Room [OR]/1-4 h after trauma) and 6 (32%) fatalities as late death (> 1 week after trauma). Four (21%) patients survived between 4 h and 1 week, thus not being classifiable in Trunkey's scheme (Figure 2). Median time interval between traumatic incident and admission to the ER was 1:02 h, and median survival time from the traumatic incident was 5:35 h.

4 patients from the early death group were brought to the ER undergoing continued CPR or necessitated CPR during immediate ER course, i.e. limiting emergency diagnostics, and thus the clinical cause of death was essentially determined by means of basic emergency diagnostics and physical examination. 3 of these patients were forensically regarded as incongruent concerning the cause of death (1 with obvious differences, 2 with marginal differences).

In summary, 5 fatalities presenting discrepancies at autopsy (3 with obvious differences and 2 with marginal differences) were remarked as early death and 1 with marginal differences as late death. Obvious und marginal discrepancies concerning the cause of death mostly occurred in the early phase of treatment (n = 5/83%); 3 of the 6 discrepant fatalities arrived at the ER undergoing continued CPR or necessitated CPR in immediate clinical course.

In the following, we present short case reports of the incongruent polytrauma fatalities in our study group.

### Obvious differences

The three fatalities presenting obvious differences at autopsy when compared to clinical diagnoses, survived the traumatic incident between 2:10 h and 2:50 h (median 2:46 h) and reached the ER 1:19 h after accident (median). Median in-hospital survival time was 1:27 h, and 2 of these patients were brought to the ER undergoing continued CPR or necessitated CPR during ER diagnostic procedures.

### Exemplary case reports

Table 2 gives an overview of ER diagnostic procedures in the reported exemplary case reports.

**Table 2:** Overview of ER diagnostic procedures in the reported exemplary case reports.

no	sex	age	mechanism of polytrauma	ISS	survival time (hh:mm/days if appropiate)	cause of death (clinically)	cause of death (autoptically)	ER diagnostic procedures
7	m	27	traffic accident (car)	59	2:50	sTBI, lung failure, hemorrhagic shock, thorax trauma	sTBI, bilateral lung contusion, pelvic trauma	physical examination, ultrasound, thoracic + pelvic x-ray, whole-body CT

8	m	72	traffic accident (pedestrian)	43	2:46	thorax trauma, heart contusion, hemorrhagic shock	thorax trauma, pericardial tamponade	physical examination, ultrasound, thoracic + pelvic x-ray, whole-body CT

12	m	49	work place accident	75	2:10	blunt thoraco-abdominopelvic trauma	fatal bleeding due to pelvic trauma	physical examination, thoracic + pelvic x-ray

2	f	24	homicidal stab attack	75	1:00	hemorrhagic shock due to multiple thoracal stab wounds	fatal bleeding due to multiple thoracal stab wounds, bilateral hematopneumothorax	physical examination

4	m	48	traffic accident (car)	50	184 days	MOF/lung artery embolism	MOF	physical examination, ultrasound, thoracic + pelvic x-ray, whole-body CT

10	m	45	train overrun	75	2:22	sTBI/hemorrhagic shock from Sinus sagittalis superior bleeding	sTBI	physical examination, ultrasound, thoracic + pelvic x-ray, whole-body CT

### Case no. 7

The patient (m, 27 y) sustained a high velocity car accident. He arrived intubated at the ER 1:13 h after the accident where sTBI and severe blunt chest trauma with bilateral lung contusions were diagnosed (ISS: 59 points). Chest drains were applied, but artificial ventilation remained difficult (P_a_O_2 _maximum 70 mmHg and P_a_CO_2 _of 70 mmHg). The patient was brought rapidly to the OR, ER diagnostics were based on physical examination, ultrasound performance, thoracic and pelvic x-ray scans and whole-body spiral computer tomography, showing sTBI, severe chest injury, pelvic trauma (left-sided iliac wing fracture, AO/OTA type 61-A2), serial fractures of the processi transversi of the vertebral column and a small retroperitoneal hematoma. During immediate damage control surgery for thoracic trauma, the man had to be resuscitated which remained unsuccessful; total survival time was 2:50 h, in-hospital survival time accounted for 1:37 h.

Based on clinical assessments, trauma surgeons assumed as cause of death a cardiac arrest caused by severe thorax trauma with consecutive lung failure and sTBI, while autopsy reported - besides the correct clinical diagnoses - a large retroperitoneal hematoma in the rearward fat tissue and consecutive signs of hemorrhagic shock as a further potentially lethal injury. While hemorrhagic shock was also diagnosed and addressed by orthopedic surgeons, the stable pelvic fracture as well as the serial fractures of the processi transversi of the vertebral column was not seen as the main source of bleeding. According to the trauma surgeons, continued ventilation problems with hypoxia and massive hypercapnia due to severe thoracic trauma in conjunction with sTBI were responsible for death primarily: the pelvic and vertebral column trauma had already been diagnosed in the ER, but respiratory failure was given priority at admission. Although death would not have been evitable, the case was forensically considered as an obvious difference since the retroperitoneal destructions with subsequent bleeding were not regarded clinically as a relevant factor of death.

### Case no. 8

The patient (m, 72 y) was overrun by a car; 1:19 h later he was admitted to the ER. During immediate ER course, CPR efforts were necessary, and initial ISS was scored 43 points. Severe chest trauma with bilateral serial costal fractures, heart and lung contusions as well as hemorrhagic shock was diagnosed; chest drains were applied. Physical examination, X-ray scans, abdominal ultrasound and spiral computertomography were performed immediately, confirming the chest trauma and showing a hypertrophied right heart without signs of pericardial effusion. After extensive CPR, the man was pronounced dead 1:27 h after admission. Total survival time was 2:46 h; trauma surgeons blamed a hemorrhagic shock for the cardiac arrest.

Autopsy confirmed the severe chest trauma with several bilateral rib fractures as well as heart and lung contusions, but furthermore yielded a pericardial tamponade with 280 ml of blood, caused by a 10 mm traumatic rupture of the V. cava inferior at the mouth of the right atrium which was considered forensically as competitive cause of death. It could not be clarified forensically to what extent the rupture of V. cava inferior was due to the original thoracic trauma or to ponderous CPR efforts with a chest drain applied to the right pleura which was probably moved during extensive closed chest CPR: the tip of the chest drain was found to be located near the mouth of the right atrium. Despite the non-diagnosed possible iatrogenic pericardial tamponade, death was considered unpreventable because of the severity of the chest trauma.

### Case no. 12

The patient (m, 49 y) was clamped by an 1800-kg-transformator that trapped him in a hoistway. After difficult extrication by the fire brigade he reached the ER 1:45 h after the accident endotracheally intubated and undergoing continued CPR. Initial ISS was scored 75 points, and emergency diagnostics consisted of basic emergency diagnostics (physical examination, thoracic and pelvic X-ray scans) as extensive CPR was continued for another 0:25 h before the patient was pronounced dead. Survival time from the accident was 2:10 h. Based upon previous surgical examination and x-ray scans, ER physicians assumed as cause of death a blunt chest, abdominal and pelvic trauma, but autopsy solely showed - besides signs of extensive CPR - a fatal bleeding from the pelvic ring disruption with traumatic rupture of A. and V. iliaca communis dextra. Organs in thorax and upper abdomen were nearly unaltered. Although merely the pelvic injury led to death by exsanguination, the patient's demise was considered unavoidable as the blood loss already necessitated CPR on scene and the patient arrived at the ER without signs of life for a total time of 1:45 h.

### Marginal differences

In the 3 fatal polytrauma issues presenting marginal differences concerning the cause of death, the patients reached the ER 1:00 h after the accident (median). 2 patients survived in-hospital for 0:12 h and 2:22 h respectively, one patient survived for 184 days. 1 patient was brought to the ER undergoing continued CPR.

### Case no. 2

The patient (f, 24 y) was assaulted with a knife and sustained 25 stab wounds to the ventral and dorsal chest wall; she was intubated on scene and brought to the ER 48 min after the attack, necessitating continued CPR already during transport. At admission to the ER, ISS was scored 75 points, and clinical procedures focussed on continuing extensive CPR. Emergency diagnostics were solely performed by means of physical examination, chest drains were not applied. She was pronounced dead 12 minutes after admission to the ER because of at least 45 min therapy-resistant asystolia; survival time from the traumatic incident was 1:00 h. Clinical cause of death was assumed as solely hemorrhagic shock due to multiple chest stab wounds with hypothesized cardiac and aortic injuries while autopsy showed besides the severe sharp thorax trauma (multiple stab injuries of lungs, pericardium, diaphragm and liver) also a bilateral hematopneumothorax contributing to the woman's demise. Heart and aorta were unaltered. Although this was considered as marginal difference and it was clearly stated that death would not have been preventable, autopsy supported the recommendation that traumatic CPR should not be stopped until bilateral chest drains have been applied [4].

### Case no. 4

A motorcycle driver (m, 48 y) was hit by a truck; 1:12 h after the accident, he arrived endotracheally intubated at the ER where the following diagnoses were collected by physical examination, ultrasound performance, thoracic and pelvic x-ray scans as well as a whole-body CT: hemorrhagic shock, spine fracture Th12, bilateral serial costal fractures, dislocated pelvic fracture, right-sided talocalcanean joint fracture, and urinary bladder rupture (ISS: 50 points). After damage control surgery and hemodynamical stabilization, the patient was transferred to the Intensive Care Unit (ICU). The further clinical course was fraught with severe complications such as rhabdomyolysis, persisting Systemic Inflammatory Response Syndrome (SIRS), decubital ulcers, critical illness polyneuropathy, and respiratory failure. Clinical diagnostics throughout the prolonged ICU treatment period consisted of the whole bandwidth of medical possibilities. Nevertheless, the patient suffered a cardiac arrest on day 184 after admission, presenting a fulminant septic shock; CPR was carried out for 45 minutes. Clinical cause of death was determined as septic MOF with concomitant septic lung artery embolism. Autopsy confirmed besides the initial diagnoses a septic MOF as inevitable cause of death, but showed - as marginal difference - no hints for presence of lung artery embolism.

### Case no. 10

The patient (m, 45 y) was overrun by a train; at admission to the ER 1:00 h after trauma sTBI, left-sided proximal humerus fracture, left-sided open hip fracture, and traumatic amputation of the left lower leg were diagnosed (ISS: 75 points). Clinical diagnostics consisted of physical examination, ultrasound performance, thoracic and pelvic x-ray scans as well as a whole-body CT. During emergency neurosurgical intervention the patient had to be resuscitated; these efforts remained unsuccessful. Total survival time from the traumatic incident was 3:22 h, in-hospital survival time was 2:22 h. With respect to timely manners and rapid aggravation of vital parameters, orthopedic surgeons blamed a bleeding from the Sinus sagittalis superior with consecutive hemorrhagic shock as the main cause of death rather than sTBI. The particular injury pattern, especially the traumatic amputation of the left lower leg did not contribute clinically to the patient's assumed hemorrhagic shock as there was no significant bleeding from the wound because of vessel retraction.

Autopsy showed - besides the correct diagnoses mentioned above - wide-spread, primarily non-survivable destructions of the entire brain tissue with comminute fractures of the basal skull and the calvarium. The cause of unpreventable death was forensically determined as sTBI, but not hemorrhagic shock, thus being qualified as marginal difference.

## Discussion

Severely injured patients present a wide range of complex problems to the trauma leader in charge: diagnosis of life-threatening injuries and their therapy need to be set up rapidly. Depending on the severity of the trauma, and thus on the time available for establishing the diagnosis, the use of diagnostic means may be very limited. Yet, a recent study points out that the in-hospital survival time is inversely proportional to the discrepancy rate at autopsy, suggesting that lack of time for appropriate diagnostic tests have an important role in missed diagnoses [18]. Especially in fatal polytrauma cases the clinically assumed causes of death might vary from the autopsy point of view, depending on several factors such as the patient's pre-existing condition and age, mechanism of accident, necessity of CPR, survival time, and the possibility to perform emergency diagnostics: if severe injury patterns with elevated ISS levels necessitate continued CPR on the patient in immediate clinical course, intended advanced diagnostic measures (besides standardly conducted performance of physical body exploration, thoracic and pelvic x-ray scans as well as abdominal ultrasound examination) usually will initially be blocked. Also in cases of immediate and/or early deaths without necessity of CPR, recommended clinical diagnostic procedures, e. g. whole-body-CT [14], cannot be carried out and diagnostic imaging might be incomplete at occurrence of death. Even with delayed deaths, numerous simultaneously uprising complications, e.g. MOF, lung embolisms, Adult Respiratory Distress Syndrome (ARDS) or other competitive causes of death can as well provoke clinical uncertainness concerning the patient's demise. In connection with clinical records autopsy may answer such questions and clarify the following points:

• estimation of manner and cause of death,

• accuracy/quality assurance of clinical diagnoses, and

• verification/denial of causal relationship between trauma incident and death.

Besides that, the cause of the traumatic incident can be clarified by autopsy in some cases, e.g. myocardial infarction, apoplectic insult or other severe pre-existing or acute diseases. Yet current literature has documented a decrease in autopsy frequency; the reasons are diversified, including technological advances in clinical testing and imaging and apprehension of potential medicolegal consequences resulting from discrepant findings. Nevertheless, disparity rates between clinical and post-mortem diagnoses concerning the cause of death are reported to reach up to 17.2% [18].

In case of trauma-related death, diagnostic assessment in polytrauma patients might also be limited at autopsy, notably in patients requiring CPR following thorax trauma: iatrogenic CPR-injuries might be sometimes hard to differentiate from the original trauma incident - cardiac massage must be considered also as a blunt chest trauma [19]. Here intensity and duration of CPR correlates with the frequency of CPR-related injuries, possibly superimposing incident-related chest trauma. In addition, usual autopsy findings in cases of trauma death need to be related to clinical data as they are not always specific for the determined cause of death, e.g. so-called "hemorrhage bleedings" (extensive subendocardial bleedings in the left ventricle) might result either from death by exsanguinations or hypotension induced by central dysregulation, e.g. sTBI [20], and lung oedema will occur as a frequent sequel of either artificial respiration or also death by central dysregulation. Furthermore, autopsy findings are also restricted in patients after explantation for organ donation purposes when explanted organs cannot be adjudged. Although performance of autopsy is currently accompanied by several additional diagnostic means, difficulties remain.

Each diagnostic procedure has limitations depending on accessible data, time available for clinical diagnostics and incident-referred pattern of trauma, and no overall diagnostic forensic and clinical gold standard is to be defined or one diagnostic measure is considered to be superior to other diagnostic measures. When executing a data-based comparison of diagnostic accuracy between clinical and forensic findings, we conclude that final statement concerning the cause of death in early polytrauma death should be conducted in synopsis of all available methods including autopsy data. A single case analysis should be comprehended in each incongruent polytrauma fatality and is necessary for accuracy in determining the cause of death.

## Limitations

We conducted a single centre study from a Level I trauma centre with a relatively small patient collective; generalization of our results may be difficult, because the management of severely injured patients in level I trauma centres might differ considerably from nontrauma centre hospitals. Furthermore, the temporal distribution of trauma deaths depends on the time intervals and classifications chosen in the given study. However, we consider our results as reliable, because review of the clinical records and death certificates as well as the final autopsy reports allowed a tight follow-up of each fatal outcome in our study by close personal interaction between surgical and forensic department. Verification of the study hypothesis was supported by the collected data. Although limitations have to be considered, we were able to demonstrate that unexpected findings concerning the cause of death are identified relatively frequently at autopsy in early polytrauma fatalities.

## Conclusion

Although preventable deaths (in terms of missed and thus non-addressed injuries) were not present, our study confirms that autopsy remains an important quality assessment tool for determining discrepant diagnoses concerning the cause of death in polytrauma fatalities, especially when autopsy is preceeded by a short ER stay and necessity of CPR during transport or in immediate ER course. A forensic autopsy in fatal polytrauma issues should standardly be conducted at least in each case of early/immediate death to help establishing the definite cause of the patient's demise - most likely already by proposal by the clinical physician in charge of the polytrauma patient. Autopsy provides important supplemental diagnosis, and inclusion of autopsy data from such polytrauma fatalities in national trauma registries for further research projects on this topic should be considered. Amplified interlocking between surgical and forensic departments ought to be strived for in the future.

## Competing interests

There is no conflict of interest. The corresponding author affirms that he has no relationships with a company whose product is mentioned in the article or with one that sells a competitive product. The presentation is impartial, and the content is independent of commercial influence.

## Authors' contributions

CTB, PG and JKF conceived of the study, and participated in its design and coordination. MT and WE participated in the design of the study. CTB drafted the manuscript. All authors read and approved the final manuscript.
